# Effects of Airborne Nanoparticles on the Nervous System: Amyloid Protein Aggregation, Neurodegeneration and Neurodegenerative Diseases

**DOI:** 10.3390/nano10071349

**Published:** 2020-07-10

**Authors:** Anna von Mikecz, Tamara Schikowski

**Affiliations:** IUF—Leibniz Research Institute for Environmental Medicine gGmbH, Heinrich-Heine-University, 40225 Duesseldorf, Germany; Tamara.Schikowski@IUF-Duesseldorf.de

**Keywords:** air pollution, Alzheimer’s disease, amyloid, *Caenorhabditis elegans*, COVID-19, dementia, neurotoxicology, particulate matter, serotonin, tire wear

## Abstract

How the environment contributes to neurodegenerative diseases such as Alzheimer’s is not well understood. In recent years, science has found augmenting evidence that nano-sized particles generated by transport (e.g., fuel combustion, tire wear and brake wear) may promote Alzheimer’s disease (AD). Individuals residing close to busy roads are at higher risk of developing AD, and nanomaterials that are specifically generated by traffic-related processes have been detected in human brains. Since AD represents a neurodegenerative disease characterized by amyloid protein aggregation, this review summarizes our current knowledge on the amyloid-generating propensity of traffic-related nanomaterials. Certain nanoparticles induce the amyloid aggregation of otherwise soluble proteins in in vitro laboratory settings, cultured neuronal cells and vertebrate or invertebrate animal models. We discuss the challenges for future studies, namely, strategies to connect the wet laboratory with the epidemiological data in order to elucidate the molecular bio-interactions of airborne nanomaterials and their effects on human health.

## 1. Introduction 

Airborne particles constitute a threat to human health. Especially, the nanosized particle fraction that is highly abundant in the urban atmosphere has the ability to penetrate virtually all organs and possesses high bioreactivity. They have also been linked to respiratory viral infections such as the SARS-CoV-2 virus and influenza as well as other respiratory and cardiovascular diseases. Recent work links combustion- and friction-derived air pollution nanoparticles (airNPs) not only to adverse health effects of the respiratory and cardiovascular systems, but also to neurodevelopmental and cognitive impairment. Consistent with this idea, this review focuses on the aging nervous system as a target of airNPs. This particularly includes novel findings concerning neurodegenerative bio-interactions of traffic-related nanomaterials in the invertebrate animal model *Caenorhabditis elegans*, which possesses a simple yet highly informative nervous system and can be investigated over its entire lifespan (i.e., enables the whole-life investigation of chronic exposures to pollutants). Medium-throughput analyses of the roundworm *C. elegans* cultivated in 96-well microtiter plate formats allow the exploration of diverse nanoparticles and their properties, including those pre- and post-use. Direct collaborations between model organism researchers and epidemiologists are suggested to identify cellular pathways of neurotoxic airNPs and thereby promote the neurosafety of nanomaterials.

### 1.1. Potential Association of Combustion- and Friction-Derived airNPs with Neurodegenerative Aggregation Diseases such as Alzheimer’s Disease

The exact mechanisms of neuronal death in neurodegenerative diseases such as Alzheimer’s disease (AD) and Parkinson’s disease (PD) are largely unknown. Studies on air pollution exposure with cardiovascular and cerebrovascular diseases suggest a harmful impact on the brain and cognitive processes through vascular and inflammatory mechanisms [1] However, the extent to which air pollution can affect cognitive decline and dementia in the elderly is not fully understood. This is despite the fact that AD as well as PD represent a growing health problem in the aging population globally. 

From the set of existing explanatory models, there is compelling genetic evidence for the aging and functional loss of protein homeostasis in cells of the central nervous system (CNS) that contributes to degenerative phenotypes. A disturbed balance between protein synthesis, folding, and degradation induces the abnormal protein aggregation in neural cells that can go as far as the formation of toxic oligomers and amyloid protein structures [2,3]. These amyloid structures are characterized by insolubility that above a certain threshold is refractory to the cellular protein degradation pathways. Amyloid protein aggregation represents a common feature of the neuropathology in AD and PD, and is closely associated with the expression of amyloid-β peptide, tau protein and α-synuclein, respectively.

In addition to aging as a risk factor for the induction of AD and PD, the contribution of environmental factors such as certain pollutants is given consideration. While case and epidemiologic studies link the premature onset of PD with pesticides or cohorts of occupationally exposed welders [4], AD has recently been correlated with urban air pollution, specifically particulate matter (PM) [5,6,7]. A meta-analysis of four significant cohorts in Great Britain, Canada, the USA and Taiwan revealed a positive association between the exposure to air pollution PM and dementia (e.g., AD). The Canadian study showed a positive association between a person’s domicile located within 50–300 m of a busy road and newly diagnosed cases of dementia with a hazard ratio of 1.12 and a 95% confidence interval of 1.10–1.14 [8]. Notably, previous studies identified the key exposure zone of traffic-related nanoparticles within 500 m and critically within 50 m from the traffic route [9,10]. The inhalation of air pollution and diesel exhaust was shown to induce inflammatory changes as well as hallmarks of AD, including amyloid formation [11,12,13] (*1.2*). A recent review summarized the results from epidemiological studies indicating that exposure to air pollution can have adverse effects on cognitive decline and impairment [14].

A new and emerging angle of urban air pollution and its adverse health effects is the contribution of rising temperatures due to climate change, especially in cities. Metropolitan areas represent vulnerable targets of the climate crisis since their buildings and pavements absorb sunlight and raise local temperatures, which in turn promote the phenomenon of urban heat islands [15]. Additionally, climate change promotes an urban microclimate that is characterized by the increase of extreme events such as the number and duration of heat waves [16]. As we know little about the interactions between a heated urban microclimate and the adverse health effects of traffic-related nanoparticulate air pollution, respective analyses assessing the role of temperature in the promotion of adverse health effects of airNPs such as neurodegeneration, that is, neurodegenerative diseases, are much needed.

### 1.2. Entry Portals—Where airNPs Have Been Found

Consistent with the idea of the urban atmosphere as a risk factor for dementia and AD, post-mortem brain samples from clinically healthy humans and dogs exposed to lifetime air pollution while living in the metropolitan areas of Mexico City or Manchester (UK) displayed typical hallmarks of AD pathogenesis, that is, aberrant deposition of amyloid-β peptide and tau protein [17]. Moreover, electron microscopy and magnetic analyses identified the presence of metal-bearing NPs, including mixed Fe^2+/^Fe^3+^ (magnetite), that represent specific combustion emissions. Of particular concern is the association of air pollution, combustion- and friction-derived NPs in young populations (i.e., children and young adults living in major cities, [6]). Rodent animal models of urban nanoparticulate air pollution show the consistent induction of inflammatory responses in major brain regions, increased DNA damage in cell nuclei of central neurons, and increased levels of AD-related tau phosphorylation [11,13].

### 1.3. Combustion- and Friction-Derived airNPs

**Figure 1:**
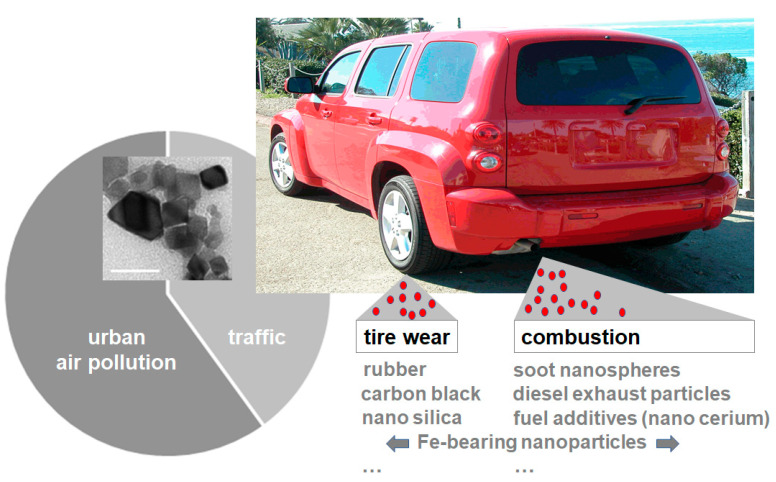
Schematic of particulate matter (PM) emissions. Traffic-related particle emissions constitute a significant proportion of urban air pollution (pie chart). Air pollution nanoparticles (airNPs) include combustion-derived nanomaterials, brake wear and tire wear. Nano cerium, for example, is used as a fuel additive in diesel vehicles (inset; representative transmission electron microscopy; bar 50 nm). For more information on airNPs, their abundance and their morphology by transmission electron microscopy, see [17].

Traffic-derived emissions are a major source of urban PM, constituting up to 80% of airborne concentrations of PM in the urban environment ([18]; Figure 1). A recent review by Gonet and Maher gives a comprehensive summary of the generation, composition and environmental distribution of transport-related particle pollution [17]. The paper likewise displays transmission electron microscopy micrographs of the most prevalent nanomaterials that are generated by fuel combustion or tire and brake wear. Air pollution nanoparticles originate from exhaust emissions such as diesel, gasoline and kerosene, but likewise from brake and tire wear [17]. Certain airNPs such as nano-sized silicon dioxide particles (nano silica) constitute both brake and tire wear [17]. Car tires profit from the addition of silica nanomaterials with regard to enhancement and durability. In contrast, NPs such as nano ceria specifically surface in diesel vehicle exhaust, due to their application as fuel additives ([19]; Figure 1, inset). In bench tests, the addition of nano ceria reduced diesel exhaust emissions of CO_2_, CO and total particulate mass in a ceria-concentration-dependent manner; however, emissions of other pollutants such as NOx (+9.3%) and the fraction of highly bioactive nanoparticulate particles (+32%) were simultaneously increased. This clearly shows that airNPs may not only pose a health hazard as a result of their intrinsic bio-interactions, but likewise via increasing the bioavailable concentrations of other traffic-related pollutants during the combustion process.

A significant increase in the airNP fraction in fuel emissions is an important health issue since animal models identified the efficient translocation of nano-sized manganese oxide particles to the central nervous system (CNS) through the olfactory bulb [20]. The entry route via the olfactory neuroepithelium (i.e., anterograde transport along axons of olfactory sensory neurons across the blood–brain barrier to the CNS) is generally used by certain spherical nanoparticles such as viruses [21,22,23], explaining both the detection of airNPs in postmortem brains and the associated amyloid neuropathology [17].

Highlighting another important issue, work by Dale et al. demonstrated that the properties of nano ceria differ before and after the combustion process [24]. The consequence of these findings is that comparative nanotoxicological studies of traffic-related airNPs are required that include engineered nanomaterials before and after use—here, collected brake or tire wear and exhaust emissions. Thus, the roadside collection of real-life particle fractions represents an informative tool to investigate the bio-interactions of airNPs. Especially, the comparative analysis of defined pre-use and (inevitably mixed) post-use nanomaterials has the potential to elucidate the mechanistic pathways of potential adverse health effects. Generally, air pollution by traffic is not only composed of airNPs, but represents a plethora of chemicals that is appropriately described as “an exploded pharmacy”. These considerations unavoidably augment to a great number of required experiments that take into account (i) the different nanomaterials composing the airNP fraction, (ii) the definition of biophysical NP properties pre- and post-use, (iii) combinations of airNPs with other traffic-related pollutants and (iv) environmental conditions such as urban climate (e.g., temperature). 

The current challenge is to develop viable research strategies that allow for comparative low- to high-throughput investigation in order to include a great variety of contributing environmental factors.

### 1.4. NP-Induced Amyloid Protein Aggregation In Vitro, Cell Culture and the Animal Model *Caenorhabditis elegans*

In previous work, it was shown that certain nanomaterials, including airNPs, induce amyloid protein aggregation and neurodegeneration using diverse research platforms such as in vitro, cell culture and invertebrate animal models.

**In vitro.** A seminal paper showed that certain nanomaterials such as copolymer particles, nano cerium, quantum dots and carbon nanotubes enhance the nucleation of protein fibrils of β2-microglobulin in the test tube [25]. These findings corroborated observations that certain surfaces of lipid bilayers, collagen fibers and polysaccharides promote the formation of amyloid fibrils. The concept developed that the unique surface area of nano-sized particles offers a biophysical environment that determines if a nanomaterial catalyzes or inhibits amyloid fibrillation of intrinsically aggregation-prone proteins [26]. The specific interactions between NP surfaces and fibrillation-prone proteins are exploited by engineered nanomaterials such as coated gold NPs that can be used as labels for amyloid fibers in postmortem brains of patients with AD and in other human tissue [27].

**Cell culture.** The analysis of cultured epithelial cells revealed that nano silica is efficiently taken up into single cells via endocytosis and reaches the cell nucleus within a few seconds [28]. In the cell nucleus, the specific surface area of silica NPs promoted the fibrillation of nuclear proteins to amorphous aggregates that grew over time to amyloid structures [29,30]. Nano-silica-induced intracellular amyloid was located by the amyloid dyes Congo red and ThioflavinT as well as amyloid-specific antibodies [31,32,33]. Investigation of the proteolytic pathways revealed that the ubiquitin-proteasome system is likewise located in silica-NP-induced amyloid aggregates and possesses proteolytic activity [32].However, this proteolytic activity is not sufficient to dissolve the aggregates. Instead, the nano-silica-induced amyloid seems to be irreversibly insoluble already. Notably, nuclear aggregates generated by silica NPs showed a similar protein composition and analogous biochemical properties to respective pathological amyloid aggregates in neurodegenerative diseases such as AD, PD and Huntington’s disease (HD) [29,32].

To summarize, nano silica that likewise constitute airNPs (i.e., brake and tire wear) represent a considerable environmental hazard because in cultured epithelial cells and neurons they induce aberrant protein fibrillation in the cell nucleus that constitutes a pathology resembling the one seen in neurodegenerative aggregation diseases and ataxias [29,32,34]. However, nano-silica-induced amyloid protein fibrillation is not confined to the cell nucleus. The formation of cytoplasmic inclusions, including the aggregation of β-synuclein interacting protein, was observed in neural cell culture and primary cortical or dopaminergic neurons exposed to silica-coated magnetic nanoparticles [35]. The paper highlights the enhanced vulnerability of neurons to the adverse effects of nano silica due to higher levels of reactive oxygen species (ROS), lower proteasomal activity and decreased cell viability. Thus, a unique vulnerability of neurons to nano silica may result from less-efficient detoxification pathways [36,37].

***C. elegans*.** To learn more about the bio-interactions of silica NPs with the nervous system in an organism, further analyses were carried out in the invertebrate animal model *Caenorhabditis elegans*. The nematode roundworm *C. elegans* has a short lifespan of 2–3 weeks and is optimally suited to interrogate NP bio-interactions during a chronic, lowest observed adverse effect level (LOAEL) exposure scenario [38]. Approximately 20,000 genes encode for the nematode’s proteins, and the majority (60–80%) of human genes, including disease genes, have a counterpart/homolog in the worm [39,40]. The etiology of neurodegenerative diseases has been extensively investigated using *C. elegans* as a model organism. Consistently, *C. elegans* is used as a tool for the screening of neuroprotective compounds, some of which are running in third phase clinical trials [41].

It was shown that silica NPs enter *C. elegans* effectively via epithelial cells of the reproductive system and the gut [42]. Corroborating the previous results from cultured epithelial and neural cells, the observation of single intestinal cells revealed that silica NPs reach the cell nucleus and induce amyloid in the nucleolus. Concerning the absorption of nutrients, nano silica interferes with the uptake of di- and tripeptides from the intestinal lumen and inhibits their downstream hydrolysis to amino acids [43]. The entire peptide metabolism is disturbed, which results in dwarfism and premature aging of young worms.

**Figure 2:**
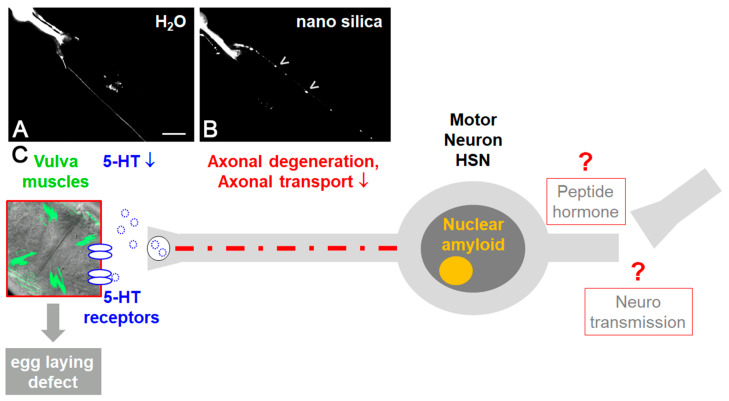
The neurotoxicity of nano silica in the neural system of the nematode *Caenorhabditis elegans*. Representative fluorescence micrographs of a single hermaphrodite-specific neuron (HSN): DsRed reporter worms of the neurotransmitter serotonin (5-hydroxytryptamine, 5-HT) were mock-treated with distilled water (**A**) or exposed to nano silica (**B**). Nano silica induced aggregation of DsRed-5-HT in the axon of the HSN neuron ((**B**), arrowheads). (**C**) Schematic of nano silica effects in *C. elegans*. Silica nanoparticles induce nuclear amyloid in single HSN neurons and protein aggregation on HSN axons. Axonal transport is disturbed and serotonergic neurotransmission at the synapse reduced. This in turn leads to defective vulva muscles and egg-laying defects [44]. Bar, 20 μm.

In single neurons, protein aggregation, neurodegeneration and defective serotonergic as well as dopaminergic neurosignaling has been observed (Figure 2; [44,45]). This is consistent with the idea that silica NPs interfere with key processes of neuronal function, ranging from nerve impulse transduction to neurotransmitter synthesis and mitochondrial energy production. The neurotoxic endpoints then induce neuromuscular defects such as reduced locomotion and paralysis (Figure 2; [46]).

However, silica NPs not only promote widespread protein aggregation and amyloid formation in wild type (N2) worms. The molecular mechanism of facilitated protein fibrillation by the specific surface area of nano silica was likewise corroborated in *C. elegans* disease models of AD, PD and HD ([42,43] Piechulek and von Mikecz, unpublished observation). In reporter worms of AD, PD and HD, hallmark proteins of the respective neurodegenerative aggregation diseases such as amyloid-β protein, tau protein, α-synuclein and poly-glutamine (polyQ) form aberrant amyloid fibrils in response to exposure to silica NPs.

### 1.5. Lifetime Exposure of Adult C. elegans to Nanomaterials

Lifespan-resolved nanotoxicology in adult hermaphrodite *C. elegans* involves cultivation in 96-well microtiter plates, where each well represents a specific microenvironment. In the presence of NPs, characteristic aging stigmata were analyzed in differently aged adult nematodes. These included (i) decrease in the rate of locomotion, swimming and pharyngeal pumping, (ii) disorganization of organ morphology (i.e., pharynx, intestine, body wall muscles), (iii) impaired protein homeostasis and increased amyloid formation or (iv) increased neurodegeneration [42,44,47]. Due to the repeated observation that certain nanoparticles reduce the health span of the worms, the concept of an aging dose (AD_50_) was introduced, which allows for the identification of toxicants that accelerate aging processes in adult *C. elegans*. The AD_50_ enables the detection of nano-sized particles that turn a young worm into an old worm [45]. While the AD_50_ has been established in *C. elegans*, an organism with a short life span, it may also be useful in long-lived individuals. Research across species, including humans, is needed to better understand the role of certain nanomaterials in aging and age-related diseases such as the neurodegenerative diseases AD and PD.

The establishment of lifetime nanotoxicology by the cultivation of adult *C. elegans* in 96-well microtiter plates represents a promising assay that allows for comparative investigations of complex airNP fractions. It enables the interrogation of urgent questions including the biophysical NP properties pre- and post-use, combinations of airNPs with other traffic-related pollutants and additional contributing environmental conditions such as urban climate (e.g., temperature). The latter can be achieved by growing the worms in liquid cultures between 15 and 25 °C.

In order to investigate how silica NPs impact molecular pathways that connect amyloid formation in single cells, global protein aggregation and neuromuscular defects of aging *C. elegans*, mass-spectrometry-based proteomics were used. It was shown that exposure of adult *C. elegans* to nano silica induced the segregation of proteins predominately involved in protein homeostasis and mitochondrial function within an SDS-insoluble aggregome network [44]. Consistently, widespread protein aggregation likewise included the axons of serotonergic hermaphrodite-specific neuron (HSN; Figure 2A). In turn, the impaired axonal transport of the neurotransmitter serotonin to the HSN synapse interrupted the neuromuscular circuit of egg laying and promoted the neural defect internal hatch (Figure 2B; [45]). Since protein aggregation in the HSN and internal hatch was rescued by anti-aging compounds that likewise inhibit amyloid formation, it was concluded that silica NPs cause premature aging in *C. elegans* by a neuropathology driven by imbalanced protein homeostasis.

## 2. Conclusions and Future Perspectives

There is increasing evidence that certain nanomaterials induce amyloid protein fibrillation in the test tube, cultured epithelial cells and primary neurons as well as diverse tissues of the animal model *C. elegans*, including the neural system. With respect to traffic-related airNPs, most studies have shown an amyloid-induction propensity of nano silica from brake and tire wear or nano ceria, which is used among other things as a diesel fuel additive.

These experimental results coincide with (i) the detection of airNPs in postmortem brains of animals and humans with chronic, long-term exposure to air pollution in metropolitan areas [6], (ii) the detection of airNPs and AD pathology in brains of laboratory mice exposed to traffic-related nanomaterials [13] and (iii) epidemiological data that identifies the proximity of a residence near busy roads as a risk factor for the development of the neurodegenerative disease AD [8].

Urbanization represents a global trend, and it is predicted that in 2050, 68% of the world’s population will live in major cities (United Nations, Department of Economic and Social Affairs, https://www.un.org/development/desa/en/news/population/2018-revision-of-world-urbanization-prospects.html, accessed 20 May 2020). To protect the health of these citizens, it seems more than reasonable to investigate potential adverse health effects of air-pollution-related nanomaterials.

How can we reconcile the data from in vitro, cell culture and animal models (i.e., the wet laboratory) with the epidemiological findings? Evidently, innovative and viable research strategies are required. We suggest identifying the molecular pathways of amyloid formation and neurodegeneration by traffic-related nanomaterials in the invertebrate animal model *C. elegans* and verifying the key components of these pathways with whole-genome analyses in humans. The option for medium-throughput, proteomics and lifespan-resolved investigation in *C. elegans* enables the comparative characterization of diverse nanomaterials, their biophysical properties pre- and post-use, diverse environmental conditions and the identification of vulnerable age groups. Key genes or pathways may be directly compared between the *C. elegans* and human genomes of individuals with long-term, chronic exposure to airNPs due to the remarkable homology between the human and *C. elegans* gene repertoires [38,39,40].

The objective is to distinguish amyloid-promoting nano-sized particles in traffic-related emissions from inactive components and thereby define neurosafe nanomaterials. An obvious question is if the nanomaterials are intrinsically neurotoxic or obtain their potential to induce amyloid formation post-use (i.e., after a high-temperature combustion process). This has consequences for potential mitigation strategies, which may include (i) the replacement of unsafe nanomaterials in tires, brakes or fuel, (ii) the amendment of combustion processes and/or (iii) ultimately, the replacement of fossil fuel combustion in motor traffic. However, rapid mitigation strategies may likewise address the identification of vulnerable target groups for adverse airNP effects in the urban population and the provision of healthier living environments by, for example, the implementation of more green infrastructure in urban planning [48].

Exposure to airNPs could also predispose exposed populations to contracting viral infections and to contracting COVID-19-associated immunopathologies. The severe acute respiratory syndrome coronavirus 2 (SARS-CoV-2) induces neurological complications, and the possible long-term impact for neurological and especially neurodegenerative diseases can only be anticipated [49]. In a worst-case scenario, the common olfactory route of SARS-CoV-2 and airNPs may exacerbate the adverse health effects on the central nervous system. Here, respective investigations are much needed.

Another important issue of future research is the premature onset of neurodegeneration by airNPs. As prenatal air pollution (i.e., diesel exhaust particles) was shown to increase anxiety and impaired cognition in male offspring of mice, the question arises if neurodegenerative defects are imprinted in early development and manifest only later in life [50]. The discussion of if and how aging processes of the neural system originate in prenatal development and the role of air pollutants has just begun.
